# Psychopharmacological characterisation of the successive negative contrast effect in rats

**DOI:** 10.1007/s00213-015-3905-2

**Published:** 2015-03-21

**Authors:** C. E. Phelps, E. N. Mitchell, D. J. Nutt, H. M. Marston, E. S. J. Robinson

**Affiliations:** 1 School of Physiology and Pharmacology, University of Bristol, Medical Sciences Building, University Walk, Bristol, BS8 1TD UK; 2 Neuropsychopharmacology Unit, Division of Experimental Medicine, Imperial College, Burlington Danes Building, Hammersmith Hospital, London, W12 0NN UK; 3 Merck Research Laboratories, Newhouse, Lanarkshire ML1 5UH UK

**Keywords:** Anxiety, Antidepressant, Affective state, Behaviour, Monoamine transmitters

## Abstract

**Rationale:**

Successive negative contrast (SNC) describes a change in the behaviour of an animal following a downshift in the quantitative or qualitative value of an expected reward. This behavioural response has been hypothesised to be linked to affective state, with negative states associated with larger and/or prolonged shifts in behaviour.

**Objective:**

This study has investigated whether different psychopharmacological treatments have dissociable actions on the SNC effect in rats and related these findings to their actions on different neurotransmitter systems and affective state.

**Methods:**

Animals were trained to perform a nose-poke response to obtain a high-value food reward (four pellets). SNC was quantified during devalue sessions in which the reward was reduced to one pellet. Using a within-subject study design, the effects of acute treatment with anxiolytic, anxiogenic, antidepressant and dopaminergic drugs were investigated during both baseline (four pellets) or devalue sessions (one pellet).

**Results:**

The indirect dopamine agonist, amphetamine, attenuated the SNC effect whilst the D1/D2 antagonist, alpha-flupenthixol, potentiated it. The antidepressant citalopram, anxiolytic buspirone and anxiogenic FG7142 had no specific effects on SNC, although FG7142 induced general impairments at higher doses. The α_2_-adrenoceptor antagonist, yohimbine, increased premature responding but had no specific effect on SNC. Results for the anxiolytic diazepam were mixed with one group showing an attenuation of the SNC effect whilst the other showed no effect.

**Conclusions:**

These data suggest that the SNC effect is mediated, at least in part, by dopamine signalling. The SNC effect may also be attenuated by benzodiazepine anxiolytics.

## Introduction

Sensitivity to gain or loss of reward has significant impact upon functioning of an individual (Wenzlaff and Grozier 1988). Generally, there is a greater sensitivity to reward loss than gain (Dreher 2007) and heightened sensitivity to loss, and failure is proposed to be a feature of negative affective states (Hajcak et al. 2004). Therefore, evaluating the sensitivity to reward loss in animals could provide an objective measure of affective state and emotion-related behaviours (Paul et al. 2005). Successive negative contrast (SNC) is defined as the behavioural response to a decrease in quantity or quality of reward (Crespi 1942). To be considered an SNC effect, performance measures e.g. response latencies or amount consumed, in response to a reduced value of the expected reward must fall below that of control animals that have only ever been exposed to the lower reward value (Crespi 1942). If the downshifted animals continue to receive the lower value of reward for several days, their performance typically returns to the level of control animals (Flaherty 1996). As such, it has been suggested that this ‘overshoot’ in response represents the emotional response to the reduced reward. This hypothesis is supported to some extent by studies investigating the SNC effect in animals exposed to modified housing conditions (Burman et al. 2008; Mitchell et al. 2012).

The SNC effect has been demonstrated using a number of different methods including consummatory (Mitchell and Flaherty 2005) and instrumental runway or lever press (Rosas et al. 2007) appetitive tasks and an aversive one-way avoidance task (Morales et al. 1992). In these tasks, the SNC effect is induced by reducing the relative value of the outcome received during sessions referred to as ‘devalue’ sessions. Previous work in our laboratory has developed and evaluated an appetitive operant chamber SNC task based on animals making a nose-poke response for reward (Mitchell et al. 2012). Similar to the five-choice serial reaction time task (5CSRTT) (Robbins 2002; Bari et al. 2008) but only using the central aperture, rats are trained to nose-poke in response to a light cue presented after a fixed intertrial interval (ITI), to receive four (baseline sessions) or one (devalue sessions) reward pellet(s). As there is only a single aperture used, the visuospatial element of the 5CSRTT is removed; however, the method still facilitates a dissociation between motivation to respond to a cue and motivation to collect reward (Robbins 2002), as well as other measures such as anticipatory responses (responses made during the intertrial interval and before the light cue) and omissions (attention). In our method, animals are trained to a stable baseline performance with the devalue session presented in a single test session and repeated once per week. This also means that animals can be tested over multiple sessions allowing for within-subject comparisons where an individual animal can be tested during both a baseline and devalue session and following multiple drug doses. We have previously shown that this approach leads to a clear SNC effect with animals in the devalue group exhibiting reduced response and collection latencies as well as increased omissions and reduced anticipatory responses (Mitchell et al. 2012). The animals also show a shift in latencies which falls below that of animals which only ever received a one-pellet reward during the same basic task (Mitchell et al. 2012).

Previous investigations have tended to show that the SNC effect is influenced by benzodiazepines (Flaherty 1990, 1996). However, the effects of antidepressants are less clear with either attenuation, facilitation or no effect on the SNC observed (Flaherty 1990; Flaherty et al. 1990; Nikiforuk and Popik 2009). To further investigate the psychopharmacology of the SNC effect, we have tested a number of compounds acting through different neurochemical mechanisms in an operant SNC task. The first set of experiments focused on compounds known to induce either anxiolytic or anxiogenic effects in humans by acting on benzodiazepine receptors or targeting the serotonin system. In addition to these compounds, we have also tested the α_2_-adrenoceptor antagonist, yohimbine, which has been shown to induce anxiety-like behaviours in animals (Cai et al. 2012). In order to understand more about the neurochemical mechanisms which contribute to the SNC effect, the final set of experiments investigated the effects of the indirect dopamine agonist, amphetamine, and mixed D1/D2 receptor antagonist, alpha-flupenthixol. The role of dopamine in the SNC effect was investigated because previous studies have found that the SNC effect is potentiated during withdrawal from the psychostimulant amphetamine (Barr and Phillips 2002).

## Materials and methods

### Animals

The subjects were two cohorts of male Lister hooded rats (*n* = 12 animals in each cohort, both Harlan, UK) weighing approximately 250 g at the start of training and 350–450 g at the start of testing. They were housed in pairs, under temperature-controlled conditions and 12:12-h reverse light–dark cycle (lights off at 0800). Rats were food restricted to maintain them at approximately 90 % of their free-feeding weights by limiting daily intake of laboratory chow to approximately 18 g per rat per day. Water was provided ad libitum. Principles of animal laboratory care were followed, and all procedures were conducted in accordance with the requirements of the UK Animals (Scientific Procedures) Act 1986 and in accordance with local institutional guidelines. All behavioural testing was carried out between 0800 and 1700 during the animals’ active phase.

### Training and testing

All behavioural training and testing was carried out using five-hole operant chambers (Med Associates, VT, USA) and controlled by K-Limbic software (Conclusive solutions Ltd, UK). Animals were trained under the schedule previously described (Mitchell et al. 2012). Briefly, training consisted of a graduated procedure similar to the one used for the 5CSRTT (Bari et al. 2008). The animals were trained to initiate the trial by making a nose-poke response in the reward magazine. After an ITI, they had to make a nose-poke response to a light cue in the central aperture within a fixed limited hold (LH) after which they could collect their reward (1 or 4 × 40 mg Noyes precision pellet, Sandown Scientific) from the magazine. Animals were trained using a seven-stage graduated procedure where each stage of training consisted of an increasing ITI, decreasing LH and decreasing stimulus duration (Mitchell et al. 2012). Training started at stimulus duration = 60 s, ITI = 2 s and LH = 60 s. Once animals had achieved criterion (>20 trials completed) for two consecutive sessions, the stimulus duration and LH were reduced, and the ITI increased to the next stage. This continued with the same criterion until ITI = 20 s, stimulus = 10 s and LH = 10 s, and they completed >20 correct trials in two consecutive sessions at this level.

Four parameters were measured: correct latency (the time between stimulus onset and correct nose-poke response), collection latency (the time from correct response to collection of food reward), omissions (failing to respond within the ITI) and premature responses (response made prior to cued stimulus). Correct trials were recorded but as all animals completed all trials in all studies, these were not analysed further. As only a single aperture was used and all animals completed all trials, only data for omissions were analysed. All animals (*n* = 12 in two separate cohorts) were trained to receive a four-pellet reward for a correct response. Both premature and omitted responses were punished with a 5-s timeout where all lights were extinguished. Following training and 10-day baseline testing with the four-pellet reward, devalue sessions were introduced in which all animals received only one pellet for a correct response. These devalue sessions were repeated once weekly (Friday) for 3 weeks in total.

During testing, animals were treated with the drug or vehicle prior to each of the session types (baseline = 4 pellets or devalue = 1 pellet). To control for non-specific effects and to facilitate comparisons between session types, each animal received all doses in a pseudorandomised study design which included randomisation of both dose and session type.

### Drug study protocol

Prior to each of the drug studies, animals were given a week baseline testing with one devalue session (devalue session on a Friday). After each drug study, the animals were given at least 1-week washout without drug treatment and at least five sessions under baseline (four-pellet reward) conditions. Each drug study was run under a counterbalanced within-subject design where each animal received each treatment prior to a baseline and devalue test session. Baseline drug testing was carried out on a Tuesday, and devalue sessions were run on a Friday with animals being run under baseline conditions without treatment on Monday, Wednesday and Thursday. Doses of drugs were based on previous SNC and 5CSRTT studies or doses shown to have anxiolytic or anxiogenic effects in other rodent tasks (Stuart et al. 2013; Torres et al. 1995; Yeung et al*.* 2013; Cai et al. 2012; Hayton et al. 2012; Cole and Robbins 1987). The first cohort of animals received a total of 16 treatments with drugs in the following order: diazepam (0.0, 0.3 and 1.0 mg/kg), buspirone (0.0, 1.0 and 3.0 mg/kg), citalopram (0.0, 0.3 and 1.0 mg/kg), yohimbine (0.0, 1.0, 3.0 and 6.0 mg/kg) and FG7142 (0.0, 3.0 and 5.5 mg/kg). The second cohort of animals was tested with the following drugs in order: amphetamine (0.0, 0.1 and 0.3 mg/kg), alpha-flupenthixol (0.0, 0.1 and 0.3 mg/kg) and diazepam (0.0, 0.3 and 1.0 mg/kg). All drugs were administered intraperitoneally with a 30-min pretreatment time except amphetamine (pretreatment 20 min) and alpha-flupenthixol (pretreatment time 60 min). Animals were returned to home cages after dosing until 5 min prior to testing.

### Drugs

Yohimbine hydrochloride (Tocris, UK) was dissolved in distilled water. Citalopram hydrobromide (Tocris, UK), buspirone hydrochloride (Sigma, UK) amphetamine (Sigma, UK) and alpha-flupenthixol (Sigma, UK) were dissolved in 0.9 % saline. FG7142 (β-carboline-3-carboxylic acid N-methylamide) and diazepam (both Sigma, UK) were dissolved in 10 % dimethyl sulphoxide (DMSO; BDH Laboratory Supplies, UK), 20 % cremophor EL (Sigma, UK) and 70 % of 0.9 % saline. All drugs were administered at a final volume of 1 ml/kg.

### Statistical analysis

All graphs were plotted using Graphpad Prism (version 5.0), and statistical analyses were performed using SPSS (version 21). Correct latency, collection latency, percent premature responses and percent omitted responses were recorded and analysed using repeated measures ANOVAs with SESSION (baseline or devalue) and DOSE (vehicle or drug at each dose) as within-subject factors. A Bonferroni confidence interval adjustment was used to compare main effects. Where significant main effects or interactions (*p* < 0.05) were observed post hoc, pairwise comparisons were made using two-tailed *t*-tests between vehicle baseline and vehicle devalue sessions and between vehicle and drug doses within each session type. Results with an alpha <0.1 were also considered as trend level effects and are discussed within the result section. A Huynh-Feldt correction was used to adjust for violation of the sphericity assumption.

## Results

### Baseline performance during the testing period

To check for stability in performance over the course of the studies, the predrug and between drug baseline data were analysed using a repeated measures ANOVA with TIME as a factor. There was no effect of TIME for correct or collection latencies or omissions (*F* < 0.78, *p* > 0.54), but there was a significant effect of TIME for premature responding (*F* (3.375, 37.127) = 3.681 *p* = 0.017) in cohort 1. Post hoc comparison did not reveal an overall trend in any particular direction (test week 1 vs test week 5 were not significantly different), but a higher degree of variability was seen with this measure. There were no effects of TIME for any of the variables in cohort 2 (*F* < 2.4, *p* > 0.12).

### Effects of acute administration of anxiolytic and antidepressant drug treatments

#### Diazepam

**Fig. 1:**
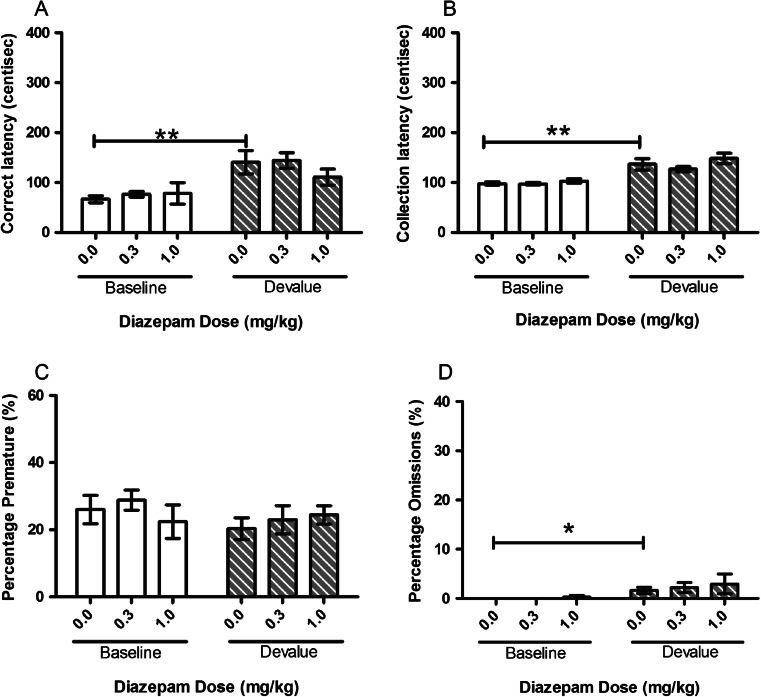
Effects of systemic treatment with diazepam (0.0–1.0 mg/kg, i.p.) on performance variables in operant SNC in the first cohort of rats tested. There was a significant main effect of SESSION for both correct and collection latencies. No significant effect of DOSE was observed on any of the measured parameters. Results are shown as mean ± SEM, *n* = 12 animals per group, within-subject, *p < 0.05, **p < 0.01 pairwise comparison between baseline and devalue session following vehicle pretreatment

**Fig. 2:**
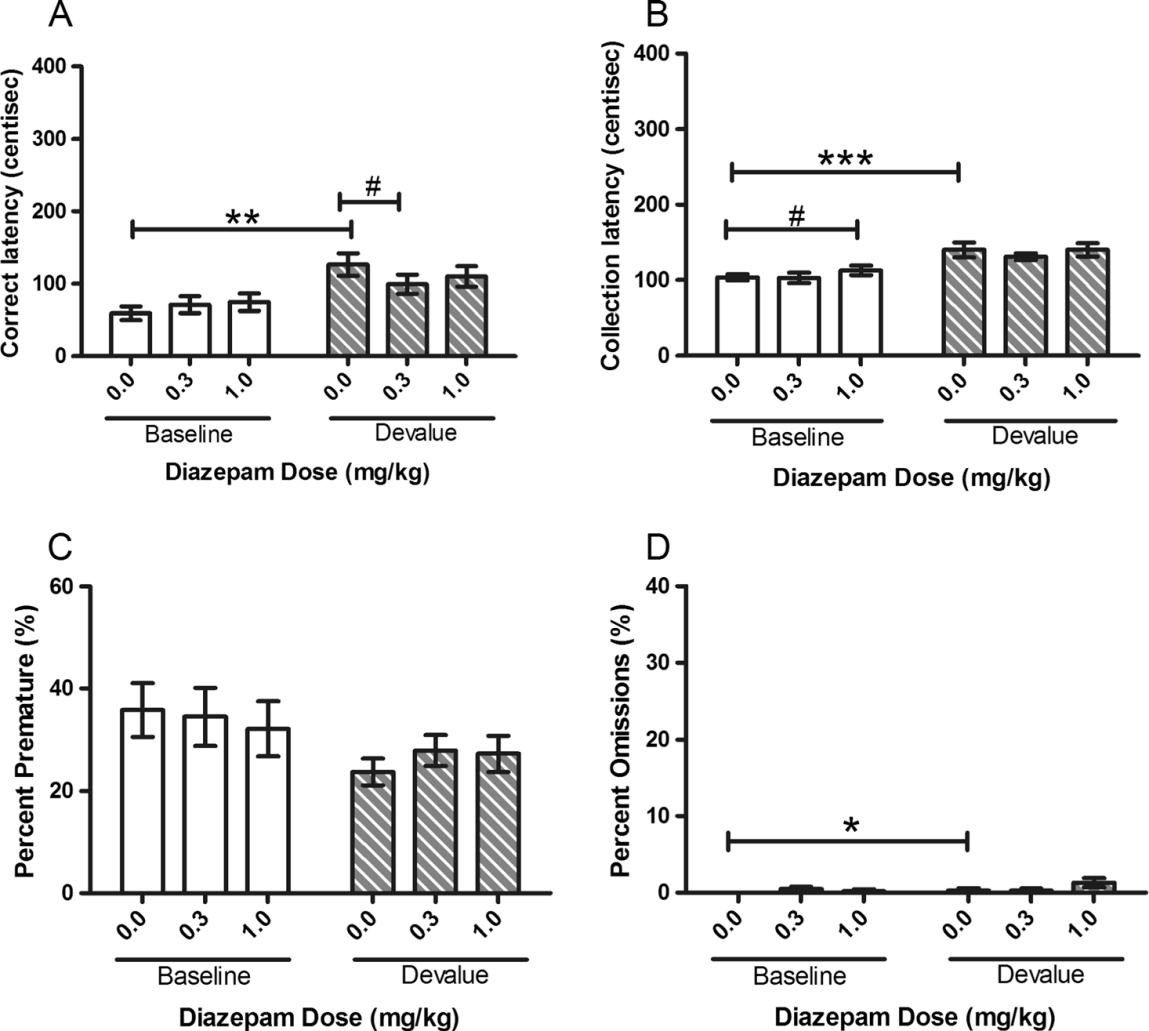
Effects of systemic treatment with diazepam (0.0–1.0 mg/kg, i.p.) on performance variables in operant SNC in the second cohort of rats tested. There was a significant main effect of SESSION for correct latency, collection latency and omissions. Diazepam (0.3 mg/kg) significantly attenuated the SNC effect on correct latency. There was no effect of DOSE on the other variable parameters. Results are shown as mean ± SEM, *n* = 12 animals per group, within-subject, *p < 0.05, **p < 0.01, ***p < 0.001 pairwise comparison between baseline and devalue session following vehicle pretreatment. #*p* < 0.05 pairwise comparisons vehicle vs drug on baseline or devalue sessions

Diazepam showed a tendency to attenuate the devalue effect. In both cohorts of animals tested, there was an effect of devaluation with a main effect of SESSION for correct latency (*F*(1, 11) = 12.23, *p* = 0.005, Fig. 1a; *F*(1, 10) = 28.872, *p* < 0.001, Fig. 2a) and collection latency (*F*(1, 11) = 38.65, *p* < 0.001, Fig. 1b; *F*(1, 10) = 66.41, *p* < 0.001, Fig. 2b). Post hoc pairwise comparisons revealed a devalue effect between vehicle-treated baseline and vehicle-treated devalue sessions with increases in both correct (*p* = 0.007, Fig. 1a; *p* = 0.001, Fig. 2a) and collection (*p* = 0.002, Fig. 1b; *p* < 0.001, Fig. 2b) latencies (Figs. 1b and 2b). There was a trend towards a main effect of SESSION in the first group for omissions (*F*(1, 11) = 4.48, *p* = 0.058, Fig. 1d) and a main effect of SESSION in the second group (*F*(1, 10) = 5.54, *p* = 0.040, Fig. 2d). Post hoc pairwise comparisons for the second group showed a significant devalue effect under vehicle treatment (*p* = 0.02).

Diazepam treatment did not specifically alter the SNC effect in the first cohort although post hoc pairwise comparison for correct latency suggested a trend towards an attenuation at the highest dose tested (1.0 mg/kg, *p* = 0.087). In the second cohort, there was no main effect of DOSE (*F*(2, 20) = 1.27, *p* = 0.302) but a trend towards a SESSION–DOSE interaction for correct latency (*F*(2, 20) = 3.34, *p* = 0.056), and post hoc pairwise comparisons revealed a significant attenuation of the devalue effect at 0.3 mg/kg dose (*p* = 0.019, Fig. 2a). There were no significant effects on collection latency or premature responses observed for either cohort (Figs. 1b and 2b, c).

#### Buspirone

**Fig. 3:**
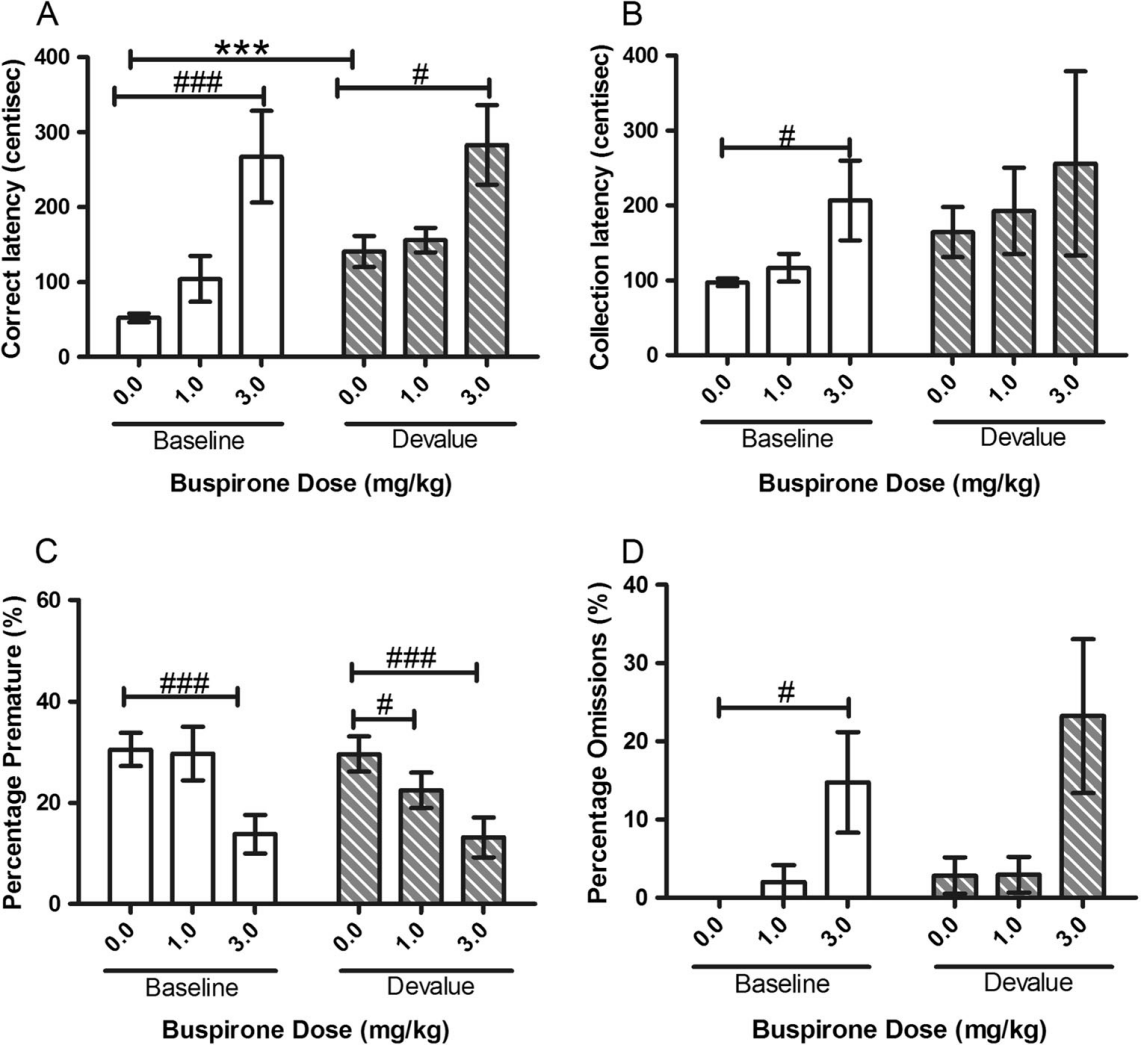
Effects of systemic treatment with buspirone (0.0–3.0 mg/kg, i.p.) on performance variables in operant SNC. No significant effects of SESSION on any variable parameter were observed, but the SNC effect was seen at vehicle for both correct and collection latencies. Buspirone (3.0 mg/kg) significantly increased correct latency in both baseline and devalue sessions. This dose significantly decreased premature responding in both sessions and increased omissions in the baseline session. Results are shown as mean ± SEM, *n* = 12 animals per group, within-subject, ***p < 0.001 pairwise comparison between baseline and devalue session following vehicle pretreatment #*p* < 0.05, #*p* < 0.01, #*p* < 0.001 pairwise comparison vehicle vs drug on baseline or devalue sessions

There were no specific effects of buspirone treatment on the SNC effect. A trend to an SNC effect was observed between baseline and devalue sessions with main effects of SESSION observed for correct (*F*(1, 11) = 3.44, *p* = 0.090, Fig. 3a) and collection (*F*(1, 11) = 4.50, *p* = 0.057, Fig. 3b) latencies suggesting a devalue effect. Post hoc pairwise comparison revealed a significant devalue effect for correct latency (*p* < 0.001, Fig. 3a) and suggested a trend towards a collection latency devalue effect under vehicle treatment (*p* = 0.055, Fig. 3b). There was no effect of SESSION for premature response or omissions. There was a main effect of DOSE on correct latency (*F*(1.504, 16.542) = 19.38, *p* < 0.001, Fig. 3a), premature responses (*F*(1.587, 17.458) = 23.70, *p* < 0.001, Fig. 3c) and number of omissions (*F*(1.205, 13.253) = 10.96, *p* = 0.004, Fig. 3d). Post hoc analysis revealed that, compared to vehicle, 3.0 mg/kg buspirone significantly slowed correct latency under both baseline (*p* = 0.003) and devalue (*p* = 0.013) conditions (Fig. 3a). Both doses of buspirone reduced premature responding in comparison to vehicle in devalue sessions (1.0 mg/kg, *p* = 0.045; 3.0 mg/kg, *p* < 0.001, Fig. 3c). In baseline sessions, 3.0 mg/kg also decreased premature responding in comparison to vehicle (*p* < 0.001, Fig. 3c). Post hoc analysis suggested that collection latency was increased at the highest dose (3.0 mg/kg) in the baseline session (*p* = 0.046, Fig. 3b). The highest dose significantly increased omissions in the baseline sessions (*p* = 0.029) and tended to increase in the devalue sessions (*p* = 0.059) (Fig. 3d). No SESSION–DOSE interaction was observed for any variable.

#### Citalopram

**Fig. 4:**
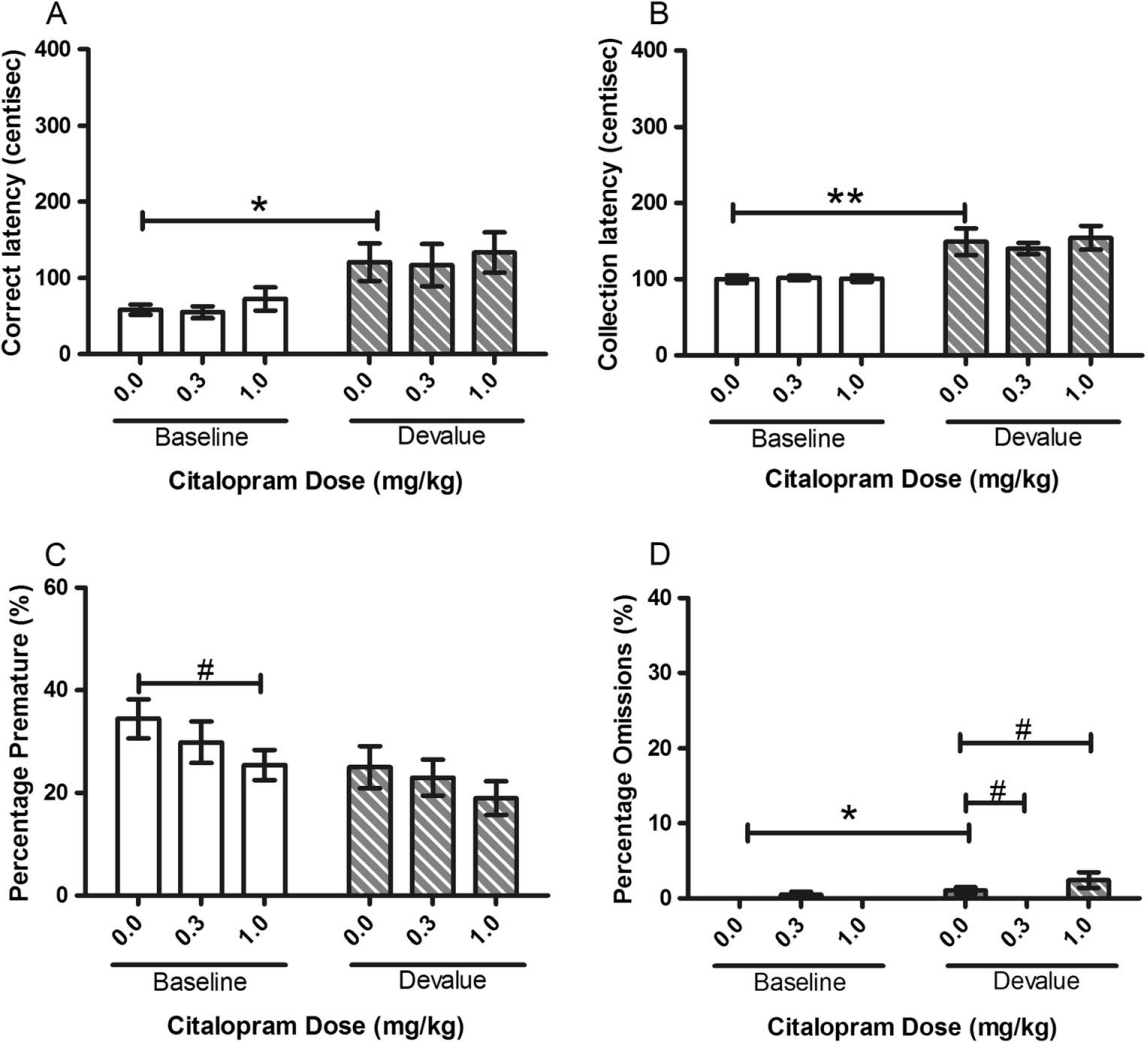
Effects of systemic treatment with citalopram (0.0–1.0 mg/kg, i.p.) on performance variables in operant SNC. No significant main effect of citalopram DOSE was seen for any of the recorded variables. A significant main effect of SESSION was seen for correct and collection latency. Results are shown as mean ± SEM, *n* = 12 animals per group, within-subject, *p < 0.05, **p < 0.01 pairwise comparison between baseline and devalue session following vehicle pretreatment #*p* < 0.05 pairwise comparison vehicle vs drug on baseline or devalue sessions

There were no specific effects of citalopram on the devalue effect. For correct latency, there was no main effect of SESSION, DOSE or SESSION–DOSE although post hoc pairwise comparison revealed a significant devalue effect for vehicle-treated baseline versus devalue sessions (*p* = 0.031, Fig. 4a). Collection latency was significantly affected by SESSION (*F*(1, 11) = 17.51, *p* = 0.002) with a devalue effect seen between vehicle-treated sessions (*p* = 0.005, Fig 4b), but no effect of DOSE or SESSION–DOSE was observed. There was an effect of SESSION for premature responses (*F*(1, 11) = 6.00, *p* = 0.032, Fig. 4c) and a trend for omitted trials (*F*(1, 11) = 4.00, *p* = 0.071, Fig. 4d) with animals showing a tendency to make fewer premature responses (*p* = 0.069, Fig. 4c) and more omissions (*p* = 0.044, Fig. 4d) on devalue sessions. There was a significant main effect of DOSE for premature responses (*F*(2, 22) = 5.93, *p* = 0.009, Fig. 4c), with a significant decrease from vehicle in the baseline session with 1.0 mg/kg (*p* = 0.028, Fig. 4c). A significant SESSION–DOSE interaction for omissions (*F*(1.104, 12.148) = 6.13, *p* = 0.027) was observed but no significant main effect of DOSE (*F*(1.120, 12.324) = 3.43, *p* = 0.085). Post hoc pairwise comparisons showed a difference in omissions between vehicle and each of the doses tested during devalue but not baseline sessions (Veh vs 0.3 mg/kg, *p* = 0.044; Veh vs 1.0 mg/kg, *p* = 0.039, Fig. 4d).

### Effects of acute administration of anxiogenic drug treatments

#### FG7142

**Fig. 5:**
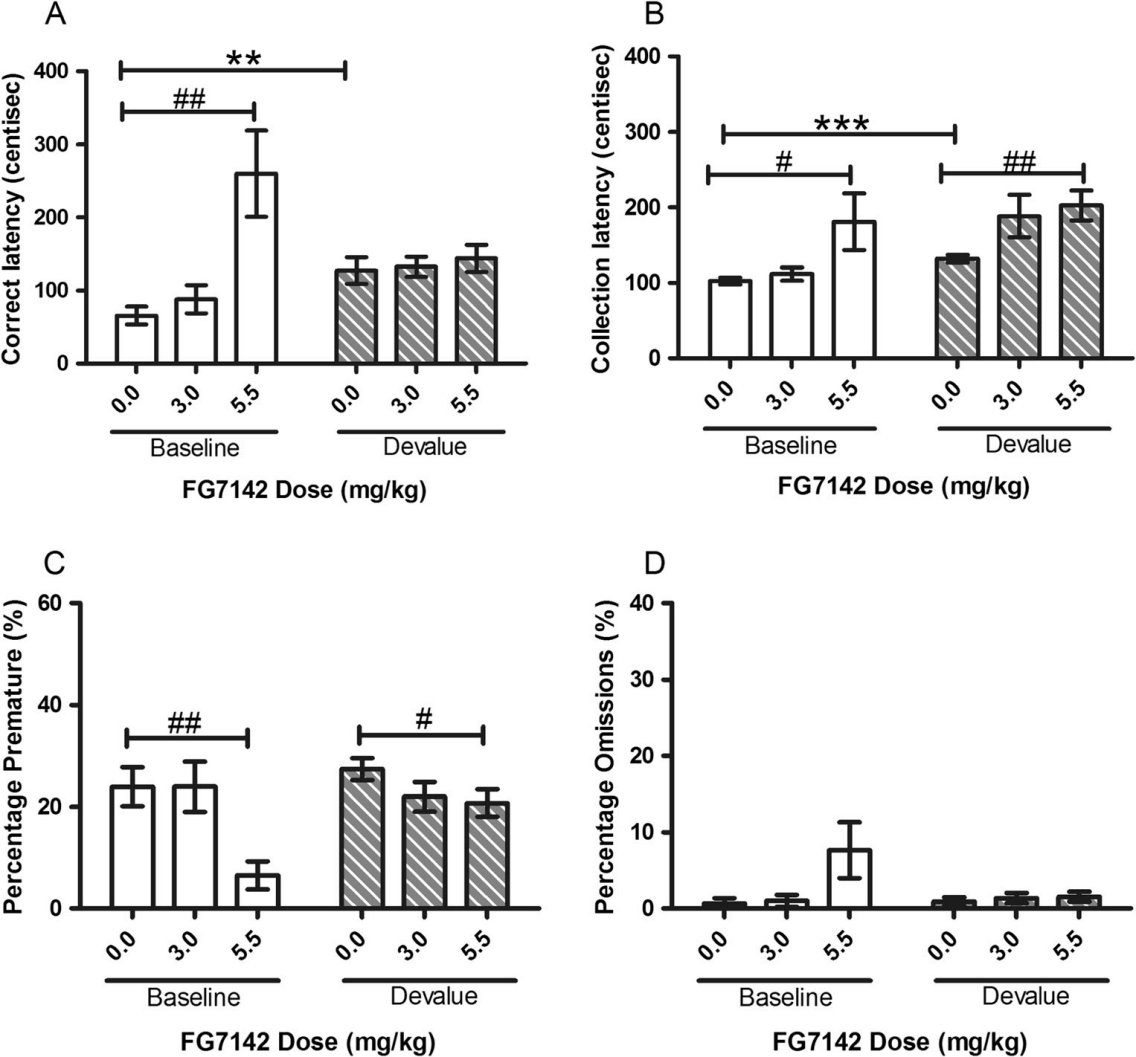
Effects of systemic treatment with FG7142 (0.0–5.5 mg/kg, i.p.) on performance variables in operant SNC. A significant main effect of DOSE was observed for correct latency and premature responding. No main effect of SESSION was seen for any of the four recorded variables. Results are shown as mean ± SEM, *n* = 12 animals per group, within-subject, *p < 0.05, **p < 0.01, ***p < 0.001 pairwise comparison between baseline and devalue session following vehicle pretreatment #*p* < 0.05, ##*p* < 0.01 pairwise comparison vehicle vs drug on baseline or devalue sessions

The highest dose (5.5 mg/kg) of FG7142 slowed collection latencies and reduced premature responses during both sessions but only increased correct latencies and omissions during the baseline session. There was a main effect of DOSE (*F*(1.373, 15.098) = 10.26, *p* = 0.003) and a SESSION–DOSE interaction (*F*(1.466, 16.128) = 9.87, *p* = 0.003) for correct latency although no main effect of SESSION. Post hoc pairwise comparisons revealed a significant devalue effect between vehicle-treated sessions (*p* = 0.009, Fig. 5a) and a slowing of correct latency at the highest dose of FG7142 during baseline sessions (*p* = 0.003, Fig. 5a). There was a main effect of SESSION for collection latency (*F*(1, 11) = 5.85, *p* = 0.034, Fig. 5b) with post hoc analysis showing a significant devalue effect at the vehicle dose (*p* < 0.001). No main effect of SESSION was observed for omissions, but there was a trend towards an effect on premature (*F*(1, 11) = 3.75, *p* = 0.079, Fig. 5c). A significant main effect of DOSE was observed for premature responding (*F*(2, 22) = 11.08, *p* < 0.001, Fig. 5c) and a SESSION–DOSE interaction (*F*(2, 22) = 3.56, *p* = 0.046). The highest dose of FG7142 caused a reduction in premature responding compared to vehicle treatment on baseline sessions (*p* = 0.002, Fig. 5c) and devalue sessions (*p* = 0.046, Fig. 5c). There was also a main effect of DOSE for omitted responses (*F*(1.217, 13.392) = 3.96, *p* = 0.034, Fig. 5d), and post hoc pairwise comparisons showed a trend towards increased omissions in the baseline session at the highest dose used (*p* = 0.056).

#### Yohimbine

**Fig. 6:**
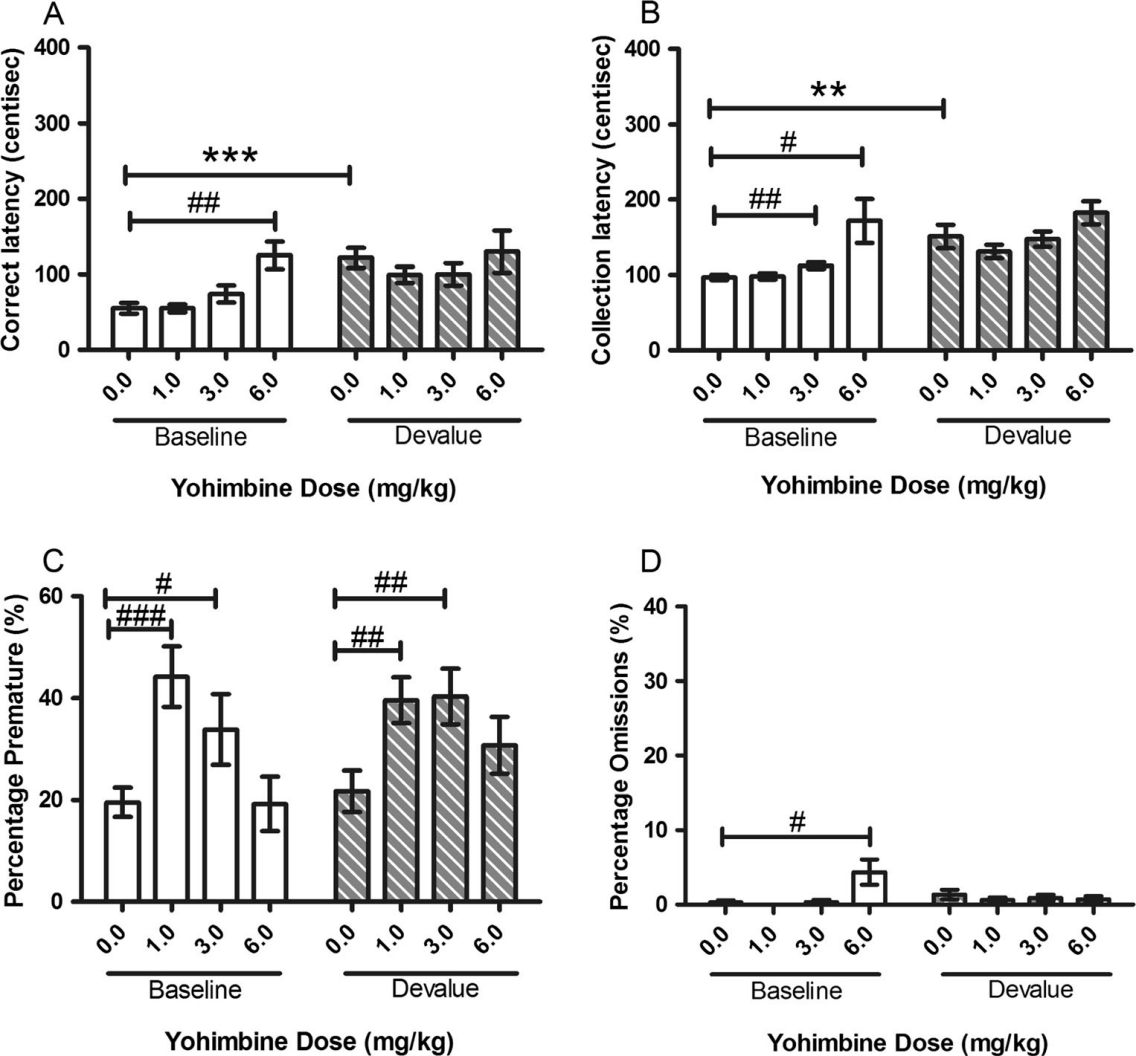
Effects of systemic treatment with yohimbine (0.0–6.0 mg/kg, i.p.) on performance variables in operant SNC. A main effect of SESSION was seen for both correct and collection latencies. Yohimbine (6.0 mg/kg) significantly increased correct latency, collection latency and omissions in the baseline sessions. 1.0 mg/kg and 3.0 mg/kg increased percentage premature in both baseline and devalue sessions. Results are shown for as mean ± SEM, *n* = 12 animals per group, within-subject, *p < 0.05, **p < 0.01, ***p < 0.001 pairwise comparison between baseline and devalue session following vehicle pretreatment #*p* < 0.05, ##*p* < 0.01, ###*p* < 0.001 pairwise comparison vehicle vs drug on baseline or devalue sessions

Treatment with yohimbine induced a dose-dependent increase in response, and collection latencies under baseline conditions but no interactions with the devalue effect were observed. At the lowest dose tested (1.0 mg/kg), yohimbine also increased premature responding during both baseline and devalue sessions. There was a main effect of SESSION observed for correct (*F*(1, 11) = 9.26, *p* = 0.011, Fig. 6a) and collection latency (*F*(1, 11) = 13.29, *p* = 0.004, Fig. 6b) reflecting an SNC effect. Post hoc comparisons showed a significant devalue effect between sessions following vehicle treatment (correct *p* < 0.001 and collection *p* = 0.006). Omissions and premature responses were not affected by SESSION (Fig. 6c, d). There was a main effect of DOSE seen for all variables: correct latency (*F*(3, 33) = 4.70, *p* = 0.008, Fig. 6a), collection latency (*F*(1.702, 18.723) = 9.89, *p* = 0.002, Fig. 6b), premature responding (*F*(3, 33) = 12.61, *p* < 0.001, Fig. 6c) and omissions (*F*(1.544, 16.987) = 4.23, *p* = 0.041, Fig. 6d). Post hoc analysis showed that the highest dose of yohimbine (6.0 mg/kg) slowed correct latency on baseline sessions (*p* = 0.004) but not on devalue sessions (*p* = 0.773). Both 1.0 mg/kg and 3.0 mg/kg of yohimbine increased premature responding compared to vehicle on both baseline and devalue sessions (1.0 mg/kg (baseline *p* < 0.001 and devalue *p* = 0.002) and 3.0 mg/kg (baseline *p* = 0.042 and devalue *p* = 0.003)), but there was no main effect of SESSION or SESSION–DOSE interaction for this variable. There was also a SESSION–DOSE interaction (*F*(1.647, 18.114) = 5.98, *p* = 0.014) for omissions, and 6.0-mg/kg yohimbine significantly increased omissions in the baseline session (*p* = 0.042).

### Effects of acute administration of dopaminergic drugs

#### Amphetamine

**Fig. 7:**
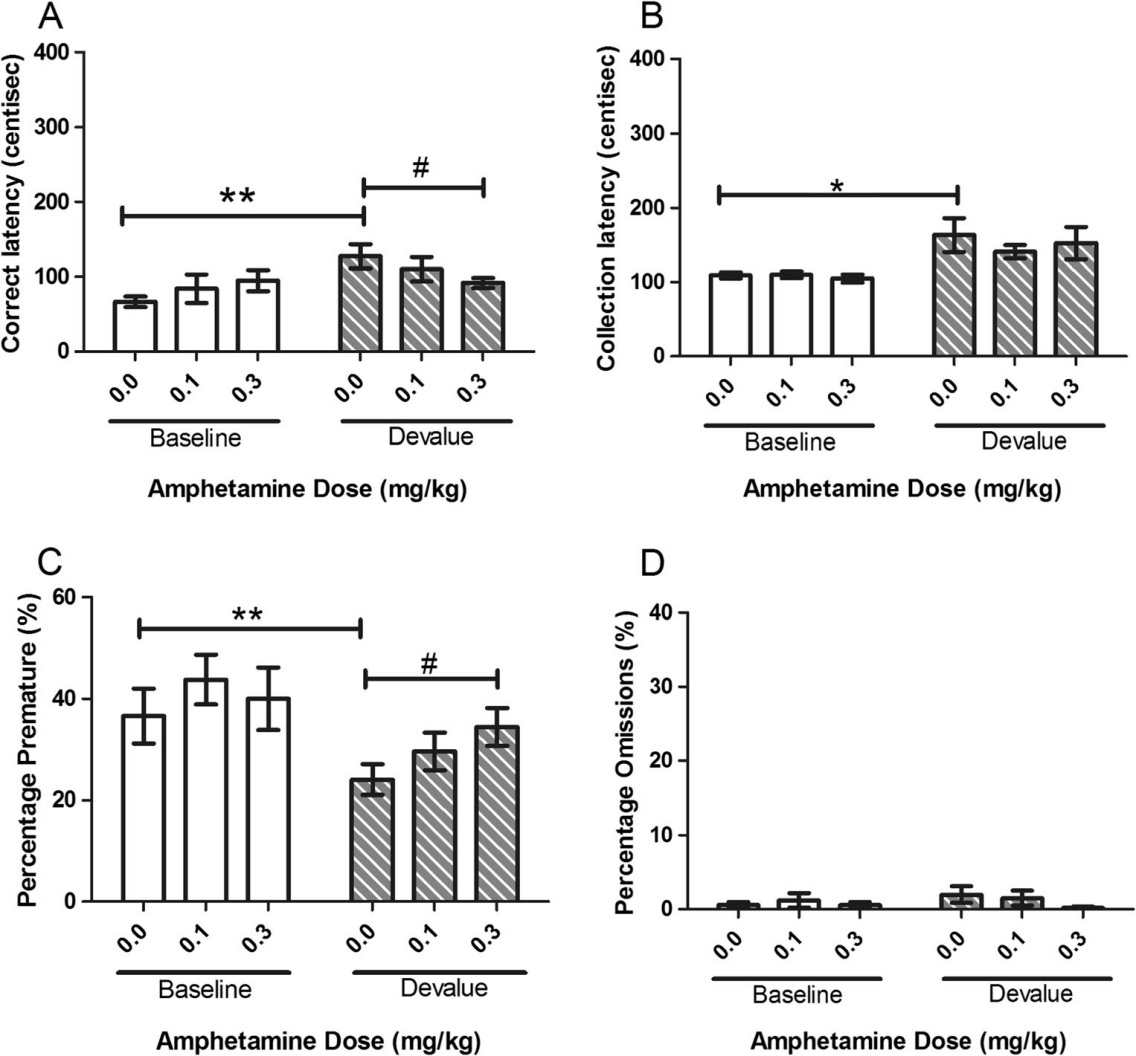
Effects of systemic treatment with amphetamine (0.0–0.3 mg/kg, i.p.) on performance variables in operant SNC. Amphetamine significantly attenuated the SNC effect on correct latency but not collection latency. There was a significant increase in premature responses in the devalue session only. No significant changes were seen with omissions. Results are shown as mean ± SEM, *n* = 12 animals per group, within-subject, *p < 0.05, **p < 0.01 pairwise comparison between baseline and devalue session following vehicle pretreatment #*p* < 0.05 pairwise comparison vehicle vs drug on baseline or devalue sessions

Amphetamine treatment induced a specific attenuation of the devalue effect on both correct latency and premature responding. There was a main effect of SESSION for correct (*F*(1, 11) = 15.68, *p* = 0.002, Fig. 7a) and collection (*F*(1, 11) = 21.33, *p* = 0.010, Fig. 7b) latencies reflecting the devalue effect. Post hoc comparisons revealed an increase in latencies during vehicle-treated devalue sessions (correct *p* = 0.002 and collection *p* = 0.030). For correct latency, no main effect of DOSE was observed; however, a SESSION–DOSE interaction was seen (*F*(2, 22) = 5.59, *p* = 0.011) suggesting a specific effect of treatment on devalue sessions. Post hoc pairwise comparisons suggested a significant attenuation of correct latency at the highest dose used (0.3 mg/kg, *p* = 0.042). No effect of DOSE or SESSION–DOSE interaction was observed for collection latency suggesting that the effects of amphetamine on the SNC effect were specific to correct latency. There was a main effect of SESSION for premature responses (*F*(1, 11) = 18.66, *p* = 0.001, Fig. 7c) but not omitted trials (Fig. 7d) and no significant effect of DOSE or SESSION–DOSE interaction on either. Post hoc analysis of the amphetamine study revealed a significant decrease in the devalue session in premature responses at the highest dose used (*p* = 0.024). Amphetamine did not have any significant effects on omissions.

#### Alpha-flupenthixol

**Fig. 8:**
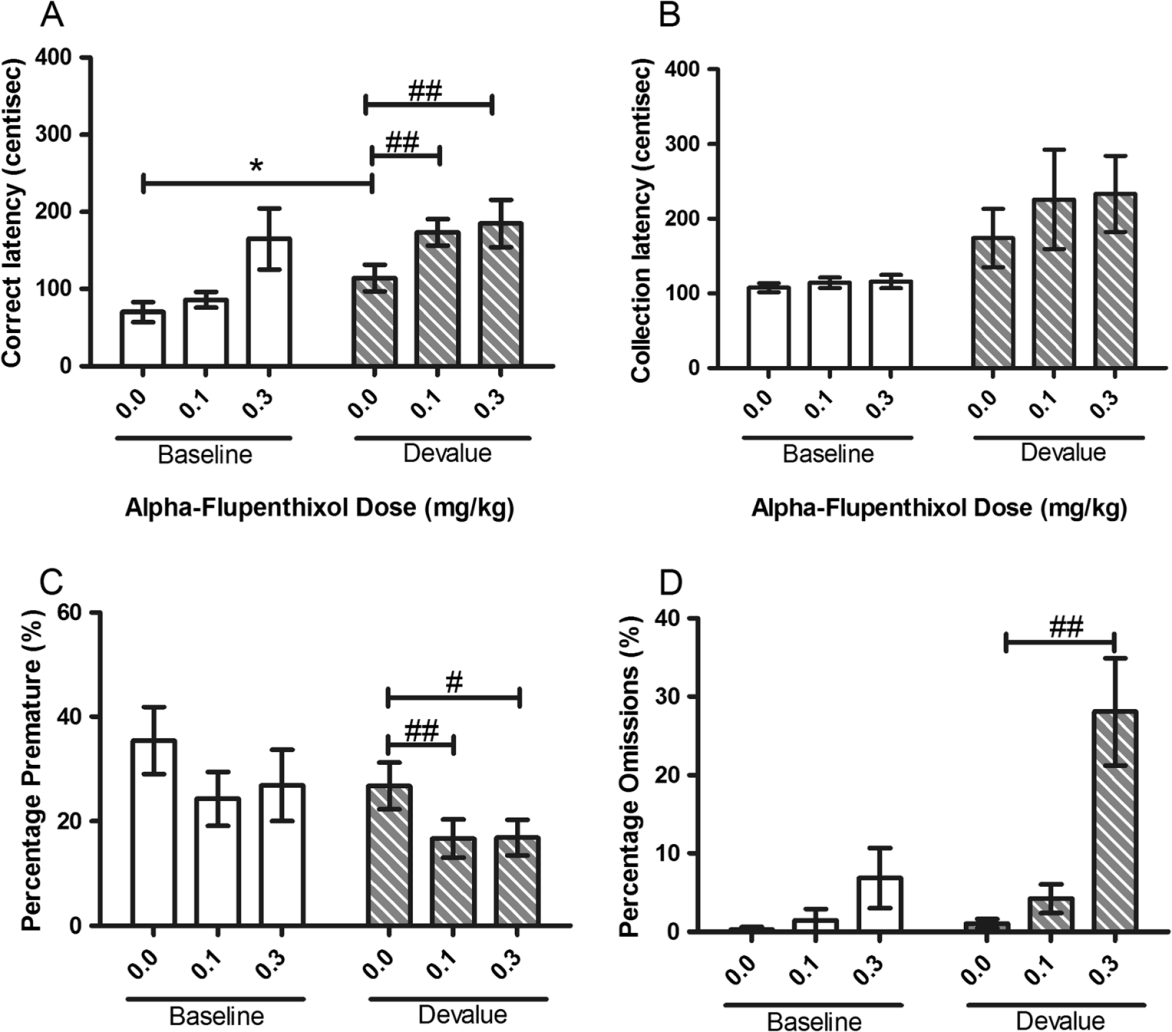
Effects of systemic treatment with alpha-flupenthixol (0.0–0.3 mg/kg, i.p.) on performance variables in operant SNC. Alpha-flupenthixol significantly potentiated the SNC effect on correct latency but not collection latency. There was a significant decrease in premature responding and an increase in omissions in the devalue session only. Results are shown as mean ± SEM, *n* = 12 animals per group, within-subject, *p < 0.05 pairwise comparison between baseline and devalue session following vehicle pretreatment #*p* < 0.05, #*p* < 0.01 pairwise comparison vehicle vs drug on baseline or devalue sessions

Treatment with alpha-flupenthixol specifically attenuated the effect of devalue on correct latency and premature responding at the lowest dose tested (0.1 mg/kg) although some more general impairments were seen at higher doses. There was a main effect of SESSION for correct (*F*(1, 11) = 8.00, *p* = 0.016, Fig. 8a) and collection (*F*(1, 11) = 12.08, *p* = 0.005, Fig. 8b) latencies reflecting the devalue effect. Post hoc comparisons revealed an increase in correct latency during vehicle-treated devalue sessions (*p* = 0.036) but not collection latency (*p* = 0.113). A main effect of SESSION was also observed for premature responses (*F*(1, 11) = 7.41, *p* = 0.020, Fig. 8c) and omitted trials (*F*(1, 11) = 16.04, *p* = 0.002, Fig. 8d). Treatment with alpha-flupenthixol potentiated the devalue effect on correct but not collection latencies. A main effect of DOSE (*F*(1.334, 14.672) = 5.95, *p* = 0.021, Fig. 6a) was observed for correct latency. Both 0.1- and 0.3-mg/kg alpha-flupenthixol increased correct latencies in the devalue session only when compared to vehicle (0.1 mg/kg, *p* = 0.002 and 0.3 mg/kg, *p* = 0.008). A trend towards increased latency at 0.3 mg/kg compared to vehicle was also seen in the baseline session (*p* = 0.056). There was no main effect of DOSE or SESSION–DOSE interaction for collection latency suggesting that the effects of treatment on the SNC effect were restricted to correct latency. There was a main effect of DOSE (*F*(2, 22) = 5.23, *p* = 0.014, Fig. 8c) for premature responses with a reduction in premature responding during devalue sessions with 0.1 mg/kg (*p* = 0.008) and 0.3 mg/kg (*p* = 0.021). There was also a trend towards attenuation of premature responding in the baseline session at 0.1 mg/kg (*p* = 0.058). A main effect of SESSION (*F*(1, 11) = 16.04, *p* = 0.002), DOSE *F*(1.207, 13.273) = 12.15, *p* = 0.003) and SESSION–DOSE interaction *F*(1.144, 12.583) = 11.64, *p* = 0.004) was observed for omissions (Fig. 8d). In the devalue sessions, 0.3-mg/kg alpha-flupenthixol increased omissions from vehicle (*p* = 0.002).

## Discussion

These data suggest that the devalue effect observed in this operant SNC task involves a dopaminergic mechanism which can be attenuated or potentiated by treatments which increase dopamine levels or antagonise dopamine receptors respectively. The relationship between drug-induced affective states and the SNC effect is less clear. Results with diazepam were inconclusive with one cohort showing an attenuated SNC effect following treatment whilst in the other group, the observed attenuation failed to reach significance. The results for buspirone and citalopram suggest a lack of effect of drugs which act at the 5-HT_1A_ receptor (partial agonism) or as a serotonin reuptake inhibitor following acute administration. However, these results do not preclude a role for other serotonin receptors or an effect if the treatments were given chronically. The anxiolytic effects of both buspirone and citalopram in the clinic are only observed following repeated administration (Goa and Ward 1986; Gorman et al. 2002). The anxiogenic effects of FG7142 and yohimbine were not associated with any specific change in the SNC effect, suggesting that drug-induced anxiety-like states do not lead to any specific potentiation of the SNC effect in this task. Interestingly, both these treatments seemed to have a greater effect during the baseline rather than devalue sessions although the reasons for this are unclear. The effects of amphetamine and alpha-flupenthixol suggest that the SNC effect on correct latency is sensitive to modulation by dopaminergic intervention treatments whilst the SNC effect of collection latency is unaffected. A similar result was also seen for diazepam in the cohort where an attenuated SNC effect was observed.

The SNC effect has previously been observed in animals performing tasks where consummatory behaviour or latency on a runway has been the main variable used to observe the devalue effect (Mitchell and Flaherty 2005; Rosas et al. 2007). This operant nose-poke task allows us to observe the devalue effect across a wider range of variables and has the potential to achieve a more comprehensive interpretation of the SNC. Consistent with our previous study (Mitchell et al. 2012), animals trained to receive a four-pellet reward showed reduced response and collection latency following devalue. In most of the studies, the number of omissions increased whilst the number of premature responses decreased, although the latter changes were less consistent. In all studies, animals continued to perform the task and completed all trials irrespective of treatment or the session. Based on previous studies using the 5CSRTT, these increases in response and collection latency most likely reflect a reduction in motivation to make a response to obtain the reduced value reward and to collect this reward once it has been delivered. The increase in the number of omitted trials also suggests effects on cognitive processes such as attention. The changes in premature responding appear to correspond with motivation for the reward as these tend to be reduced when the value is downshifted. Animals are still performing the task and completing all trials, but the impact of devaluing the outcome may also reduce their attention and therefore overall accuracy. It would be interesting to test this further using a full five-whole version of this SNC task where visuospatial attention is also tested.

Amphetamine, an indirect dopamine agonist, attenuated the SNC effect on correct latency and premature responding. In contrast, the D1/D2 antagonist, alpha-flupenthixol, potentiated the SNC effect on these variables. No effects on collection latency were observed although a clear devalue effect was present in all the studies. These results compliment findings that the devaluation of a reward decreases the dopamine response in the nucleus accumbens in consummatory SNC (Genn et al. 2004). These findings are also consistent with previous studies which have shown that animals withdrawn from the psychostimulant amphetamine show an exaggerated SNC effect, which may be related to the effects of drug withdrawal on the dopamine system (Barr and Phillips 2002). Our version of an SNC task allows distinction between drive to respond to obtain a reward (correct latency, premature and omissions) and motivation to collect a reward (collection latency). In this study, it was found that the latency to respond for the reward but not the latency to collect reward was modulated by dopaminergic manipulations during the devalue sessions. This observation corresponds with previous work using the same task with manipulations of affective state (Mitchell et al. 2012). Together with previous studies investigating dopaminergic mechanisms involved in the 5CSRTT (Robbins 2002), our findings suggest that differential mechanisms contribute to the devalue effect associated with the drive to obtain reward compared with those modulating motivation to collect the reward once dispensed. In our study, dopaminergic signalling was only involved in response latency, suggesting that the SNC effect on motivation to collect reward involves a nondopaminergic mechanism. Interestingly, previous studies have found links between the SNC effect and opioid system which is also strongly connected to the regulation of dopamine levels in the reward system in the CNS (Devine et al. 1993). The opioid agonists morphine and DPDPE both attenuate the SNC effect (Rowan and Flaherty 1987; Wood et al. 2005) whilst the opioid antagonist naloxone potentiates it (Pellegrini et al. 2005).

Serotonergic modulation did not significantly influence the SNC effect in our studies. Neither acute treatment with the selective serotonin reuptake inhibitor (SSRI) antidepressant citalopram nor 5-HT_1A_ partial agonist and anxiolyticbuspirone had a significant specific effect on correct or collection latencies. Citalopram is an anxiolytic in both conditioned freezing (Inoue et al. 1996) and shock-induced modulation (Schreiber et al. 1998), but it is an anxiogenic in the elevated plus maze (Pollier et al. 2000) and the light–dark exploratory test (Sanchez and Meier 1997). However, in this study, no specific effects were seen suggesting that our operant SNC is not sensitive to SSRIs. Results presented here also show no specific effect of buspirone in the task. The highest dose of buspirone used did affect latencies in both types of session as well as reducing premature responses and increasing the number of omissions. As these effects occurred during both trial types, they are more likely due to nonspecific effects such as mild sedation (Vaidya et al*.* 2005; Pavlakovic et al. 2009). Similar to our findings, neither acute nor chronic buspirone had any effect on SNC in either a one-way avoidance learning task (Torres et al. 1995) or in consummatory SNC (Flaherty et al. 1990).

One of the more widely studied receptor systems linked to SNC and anxiety is the benzodiazepine receptor (Flaherty et al. 1980; Morales et al. 1992; Nikiforuk and Popik 2009). However, in our study, the benzodiazepine agonist diazepam was found to either attenuate or have no specific action on the SNC effect depending on the cohort of rats. It is difficult to draw any conclusions from these differences, although diazepam was apparently more effective in the cohort that was tested at the end of the experiment after extensive training and testing. In this cohort, there was a specific attenuation of the devalue effect at 0.3 mg/kg diazepam. Similar to the dopaminergic treatments, the effect was specific to correct latency, and no effect on collection latency or other variables was observed. This dose of drug also had no effect on performance measures during baseline testing suggesting a specific interaction with the devalue effect. Previous studies have also found a benzodiazepine-induced attenuation of the SNC effect. The benzodiazepine agonist, chlordiazepoxide, has been shown to be effective from the first day of reinforcer downshift in a lever press task (Nikiforuk and Popik 2009) and in reducing contrast from the second postshift day in consummatory contrast protocols (Flaherty et al. 1980).

In terms of the relationship between increased anxiety and the SNC effect, rats bred for high anxiety exhibit a greater SNC effect in an instrumental runway task (Rosas et al. 2007). From this, it was hypothesised that pretreatment with anxiogenic drugs would potentiate the SNC effect. However, in this study and task design, the benzodiazepine inverse agonist, FG7142, and α_2_-adrenoceptor antagonist, yohimbine, did not specifically potentiate the SNC effect. The highest dose of FG7142 increased correct latency and reduced premature responding on baseline sessions only, which may be a result of lower motivation from effects of FG7142 on appetite (Cottone et al. 2007). At the highest dose of yohimbine, both correct and collection latencies were increased on baseline sessions, which may indicate an anxiogenic effect of yohimbine influencing general task performance. Interestingly, yohimbine increased premature responses during both baseline and devalue sessions which were opposite to the effect seen with FG7142. This increase in premature responding may involve a specific effect on impulse control and the proposal that α_2_-adrencoceptors play an important role in regulating this behaviour (Franowicz et al. 2002; Arnsten 2011).

In our study, we did not use a separate, non-shifted control group which may represent a potential limitation. In most previous studies investigating the SNC effect (Flahert*y* et al. 1980; Barr and Philips 2002; Genn et al. 2004; Mitchell and Flaherty 2005; Pellegrini et al. 2005; Burman et al. 2008; Nikiforuk and Popik 2009), a control group which only ever received the low-value reward was included. This control is often included for two reasons: first, to demonstrate a devalue effect defined as a shift to a level below that of animals which only ever received the lower value reward (Crespi 1942) and second, to provide a treatment control for between-subject study designs. Our previous work using this task has shown the devalue effect against a one-pellet control group (Mitchell et al. 2012), but a within-subject design for experimental manipulations, as in our current study, potentially negated the need for a lower value reward control group. One limitation of this method is that it is not possible to determine if the drugs would have shown different effects in animals which only received low- or high-value reward throughout.

Another limitation of this study may be that premature responding was not stable in cohort 1 and that premature responses were not consistently reduced during devalue. This may suggest that this measure is not the most reliable indication of the SNC effect in this task. In contrast, all other measures were stable throughout the study, and these measures form the main basis for the conclusions presented in this paper.

Together, results presented here suggest that the SNC effect is at least partially mediated by dopaminergic signalling. Dopamine is known to play an important role in reward processing, and the recognition and subsequent adaptive changes in behaviour during devalue may reflect changes in dopamine signalling (for review, see Schultz 2010). For example, studies in monkeys have shown that learning of a reward predictive cue results in firing of dopamine neurones in response to presentation of the cue. However, if the subsequent reward is not delivered, a reduction in dopamine neuronal firing is seen (Mirenowicz and Schultz 1994). Dopamine is also thought to play a role in affective behaviour with anhedonia being linked to mood disorders both in humans and animals (DSM-V; Cryan and Slattery 2007; Nestler and Hyman 2010). Although we did not see any specific effects with the anxiogenic manipulations used in this task and results with the serotonergic drugs were also negative, the results for diazepam suggest that there may be some relationship between affective state and the SNC effect. Previous studies which have shown a link between the magnitude of the devalue effects and affective state generally used long-term manipulations such as chronic mild stress (Burman et al. 2008). It may therefore be that the SNC effect is sensitive to negative affective states more akin to depression than anxiety. In our study, all drugs were administered acutely, and further studies using chronic drug treatments are needed to address this issue. It would also be interesting to look at these treatments in animals who have a depression-like phenotype such as those exposed to chronic mild stress (Cryan and Slattery 2007; Nestler and Hyman 2010).
